# Using meta-analysis and machine learning to investigate the transcriptional response of immune cells to *Leishmania* infection

**DOI:** 10.1371/journal.pntd.0011892

**Published:** 2024-01-08

**Authors:** Zahra Rezaei, Ahmad Tahmasebi, Bahman Pourabbas

**Affiliations:** 1 Professor Alborzi Clinical Microbiology Research Center, Shiraz University of Medical Sciences, Shiraz, Iran; 2 Shiraz Institute for Cancer Research, School of Medicine, Shiraz University of Medical Sciences, Shiraz, Iran; Insitut Pasteur de Tunis, TUNISIA

## Abstract

**Background:**

Leishmaniasis is a parasitic disease caused by the *Leishmania* protozoan affecting millions of people worldwide, especially in tropical and subtropical regions. The immune response involves the activation of various cells to eliminate the infection. Understanding the complex interplay between *Leishmania* and the host immune system is crucial for developing effective treatments against this disease.

**Methods:**

This study collected extensive transcriptomic data from macrophages, dendritic, and NK cells exposed to *Leishmania* spp. Our objective was to determine the *Leishmania*-responsive genes in immune system cells by applying meta-analysis and feature selection algorithms, followed by co-expression analysis.

**Results:**

As a result of meta-analysis, we discovered 703 differentially expressed genes (DEGs), primarily associated with the immune system and cellular metabolic processes. In addition, we have substantiated the significance of transcription factor families, such as bZIP and C2H2 ZF, in response to *Leishmania* infection. Furthermore, the feature selection techniques revealed the potential of two genes, namely *G0S2* and *CXCL8*, as biomarkers and therapeutic targets for *Leishmania* infection. Lastly, our co-expression analysis has unveiled seven hub genes, including *PFKFB3*, *DIAPH1*, *BSG*, *BIRC3*, *GOT2*, *EIF3H*, and *ATF3*, chiefly related to signaling pathways.

**Conclusions:**

These findings provide valuable insights into the molecular mechanisms underlying the response of immune system cells to *Leishmania* infection and offer novel potential targets for the therapeutic goals.

## Author summary

Leishmaniasis is a group of diseases caused by protozoan parasites belonging to the genus *Leishmania*. The infection involves various cells, and effective diagnosis and treatment require a clear understanding of its multiple aspects. We conducted a study using meta-analysis and feature selection algorithms combined with co-expression analysis to shed light on the key responsive genes and mechanisms involved in *Leishmania* infection. Our findings revealed transcriptional signatures associated with essential cellular metabolic functions and immune system response. We also confirmed the critical role of transcription factor families, including bZIP and C2H2 ZF, in response to *Leishmania* infection. The applied feature selection techniques identified two genes that show potential as *Leishmania* infection biomarkers and treatment targets. Moreover, our co-expression analysis identified seven crucial hub genes in signaling pathways. These findings provide valuable insights into the molecular mechanisms underlying the immune system cells’ response to *Leishmania* infection and present novel potential targets for therapeutic intervention.

## Introduction

Leishmaniasis, with the second rank in mortality and fourth in morbidity, is one of the world’s most neglected tropical diseases caused by obligate intracellular protozoan parasites of the genus *Leishmania* [1]. According to the world health organization (WHO), leishmaniasis poses a risk to 350 million people in 98 countries, with an annual incidence of almost 1.5 million new cases [2]. Different species of *Leishmania* cause various forms of the disease, including cutaneous leishmaniasis (CL), diffuse cutaneous leishmaniasis (DCL), mucocutaneous leishmaniasis (MCL), and visceral leishmaniasis (VL) or kala-azar. Immune responses against leishmaniasis are particularly complex, and their initiation and development depend on a complex range of cells. However, it is not entirely known how immune cells and the numerous mediators they produce work leading to resistance or susceptibility to leishmaniasis. [3].

Typically, macrophages can polarize to either classically activated (M1) or alternatively activated (M2) subsets in response to environmental stimuli. Reactive oxygen and nitrogen species (RONS) and the secretion of pro-inflammatory cytokines are characteristics of M1 macrophages, whereas M2 macrophages produce large quantities of anti-inflammatory cytokines and exhibit cell surface markers that aid in tissue remodeling and inflammation resolution [4].

Understanding the complex interplay between *Leishmania* and the host immune system is crucial for developing effective treatments against leishmaniasis. Previous studies have identified various molecular pathways and signaling networks involved in the immune response to *Leishmania* infection [5–7]. For instance, Fakiola et al. utilized whole blood transcriptional profiles associated with asymptomatic infection, active disease, and treated cases of visceral leishmaniasis. Through this analysis, they identified differentially expressed genes (DEGs) associated with erythrocyte function, cell cycle, interferon-γ, interleukin signaling, chemokine signaling pathways, and the Aryl Hydrocarbon Receptor signaling pathway in different stages of the disease [5]. Gonçalves and his team used gene networks to study how *Leishmania* infection affects macrophages. They discovered that the Th17 pathway is activated in the early infection stages but becomes less active as the infection progresses, which helps us understand how genes and factors control the immune response in infected cells over time. [6]. By analyzing dynamic transcriptome data obtained from *Leishmania major* infected bone marrow-derived macrophages from resistant and susceptible mice, Bouabid et al. identified DEGs that may account for the differences in the immune response to infection between the two strains [7].

The availability of transcriptomics data offers unique opportunities to evaluate fundamental mechanisms involved in the cell host response to *Leishmania* infection. Meta-analysis and machine learning are effective techniques for investigating gene profile changes in high-dimensional expression data. Meta-analysis is a valuable and robust strategy to integrate different gene expression datasets, leading to comprehensive and reliable results with increasing sample size and statistical power [8]. The meta-analysis methods, such as effect sizes, evaluate the heterogeneity among datasets and summarize scientific evidence from multiple studies by determining total scores from individual datasets and assessing them based on the statistical significance of all studies [9]. The meta-analytic techniques have been well exploited to detect the gene signatures in breast [10], pancreatic [11] cancers, and Alzheimer diseases [12]. Machine-learning techniques are efficiently used to detect gene features from complex expression datasets. Several learning algorithms, such as supervised and unsupervised-based learning, have been extensively applied to find differentially expressed, prognostic-related, and therapeutic targets [13].

**Fig 1:**
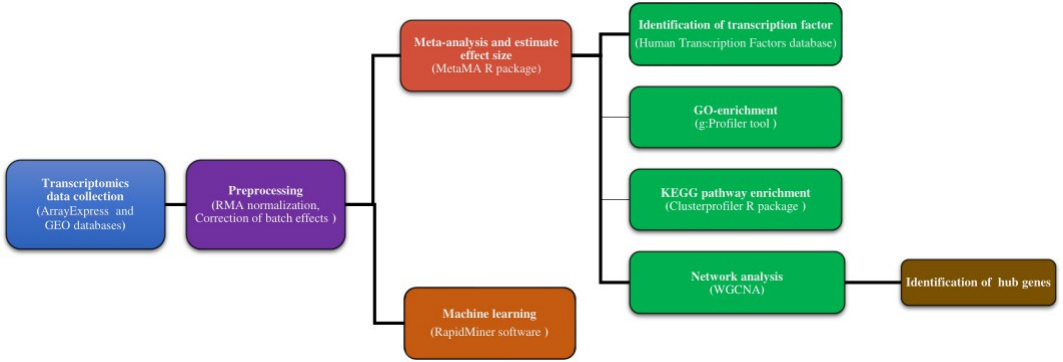
Schematic overview of the strategy for understanding aspects of response of immune system cells to *Leishmania* infection.

To obtain significant transcriptional information for how immune system cells respond to *Leishmania*, we combined several data sets from transcriptomic studies and integrated meta-analysis, feature selection algorithms, and co-expression network analysis (Fig 1). We found a list of genes and a set of modules to provide a basis for exploring molecular process responses in immune system cells. The results can be used to determine essential candidate genes and therapeutic targets for leishmaniasis and can also increase the understanding of gene-gene interactions under this infectious disease.

## Material and method

The raw data of microarray experiments were retrieved from Gene Expression Omnibus (GEO, www.ncbi.nlm.nih.gov/geo/) and ArrayExpress (www.ebi.ac.uk/arrayexpress). The keywords " leishmaniasis, *Leishmania donovani*, *Leishmania major*, *Leishmania braziliensis*, dendritic cell, macrophages", and their combinations were used for the search. The results were filtered by the organism (*Homo sapiens*), and a series was selected for the analysis if they included control subjects and patients and carried out in macrophages, NK, and dendritic cells. Affymetrix datasets were normalized and background corrected with Robust Multi-array Average (RMA) using the Expression Console software. Agilent datasets were quantile normalized, and log2 was transformed using the limma R package [14]. After pre-processing, probe IDs from different platforms were matched to their ensemble ID. For the cases where multiple probe IDs were mapped to the same ensemble ID, the probe with the greatest interquartile range (IQR) was selected. The empirical Bayes algorithm (ComBat) was used to reduce batch effects among the studies using the SVA R package [15]. In addition, studies with more than two conditions separated into sub-datasets and during meta-analysis were considered individual datasets. Finally, a meta-analysis in accordance with the effect size combination method was carried out using the metaMA R package [16] to compare diseases and healthy controls. Based on the false discovery rate, differentially expressed genes (DEGs) were determined using an adjusted p-value <0.05.

### Functional enrichment analysis

The Gene Ontology (GO) of the DEGs was obtained from the g:Profiler tool [17], and significant Gene Ontology (GO) terms were defined using an adjusted *P*-value of <0.05. REVIGO was used to reduce the redundancy of the significant GO term lists based on the default parameter with “allowed similarity” of medium (0.7) [18]. To explain the changes of metabolic pathways between diseases and healthy controls, all DEGs were subjected to Reactome pathways enrichment analysis using Clusterprofiler R package [19], and the significant pathways were collected based on statistical significance (p ≤ 0.01). In addition, The Human Transcription Factors database [20] was used to identify transcription factors (TFs) and their families.

### Construction of co-expression network

In this study, a weighted gene co-expression network analysis (WGCNA) [21] was applied to normalized expression values of DEGs to identify gene modules and screen hub genes. In summary, a similarity matrix was formed based on calculating Pearson’s correlation coefficient between any two genes. The similarity matrix was transformed into an adjacency matrix that was calculated as Aij = (|0.5 + (0.5*cor (xi, xj))|)^β^ using a soft-thresholding power (β) of 10. Next, the adjacency matrix was converted into a topological matrix that was used for the hierarchical clustering of genes. Finally, a *dynamic Tree Cut* algorithm with a minimum module size of 30 genes was performed to identify modules. The hub genes were also defined based on each module’s highest degree of connectivity. We also used the clusterProfiler R package to perform functional and pathway enrichment analysis on each module.

### Machine learning models

Machine learning algorithms were used to improve the detection of the gene expression features between diseases and health conditions. At first, gene expression values were converted to Z-standardized values, and then standardized expression values were applied to attribute weighting (feature selection) models. The input matrix was composed of 141 samples, with 89 samples representing infected cases and 52 samples representing healthy cases. Each sample was labeled as either a disease or a health instance. We utilized nine distinct attribute weighting algorithms (Uncertainty, Support Vector Machine (SVM), Chi-Squared, Information Gain, Information Gain Ratio, Deviation, Gini Index, Relief, and PCA) to identify the most important genes. These algorithms encompassed a range of techniques such as Uncertainty, which estimated attribute weight based on symmetrical uncertainty with respect to the class, and Gini Index, which utilized the Gini index of the class distribution. We also leveraged the support vector machine (SVM) algorithm, a versatile tool widely used in data analysis and pattern recognition. Specifically, we utilized the coefficients associated with the normal vector of a linear SVM to assign weights to the features. Furthermore, the weight Deviation method generated weights by considering the standard deviations of all attributes. These weights were then normalized using either the average, minimum, or maximum values of the respective attribute. Alongside these techniques, the Chi-Squared model assessed attribute importance through the chi squared statistic, while Information Gain measured attribute relevance by evaluating the information gain in class distribution. Additionally, Information Gain Ratio, a modified version of Information Gain, took into account attributes with fewer distinct values. Additionally, the weight by PCA method leveraged PCA to generate feature weights based on their relevance to the class feature. Meanwhile, Relief, an independent and efficient attribute weighting model, accounted for noise and feature interactions using random instance selection. The resulting weights from each feature selection model were normalized between 0 and 1 to ensure comparable significance. A weight closer to 1 indicated higher importance of a gene in distinguishing infected and healthy conditions, as determined by the employed feature selection model [22–25].

Moreover, to assess the accuracy of the feature weighting algorithms, we utilized nine new datasets created from the significant features identified by each algorithm. These datasets were derived from the original dataset. Decision tree models (decision tree and random forest) were applied to these nine datasets. The accuracy of the algorithms was evaluated using 10-fold cross-validation [22, 26]. The machine learning algorithms were performed with the RapidMiner software [27].

## Result

### Identification of Differentially Expressed Genes (DEGs) by meta-analysis

**Fig 2:**
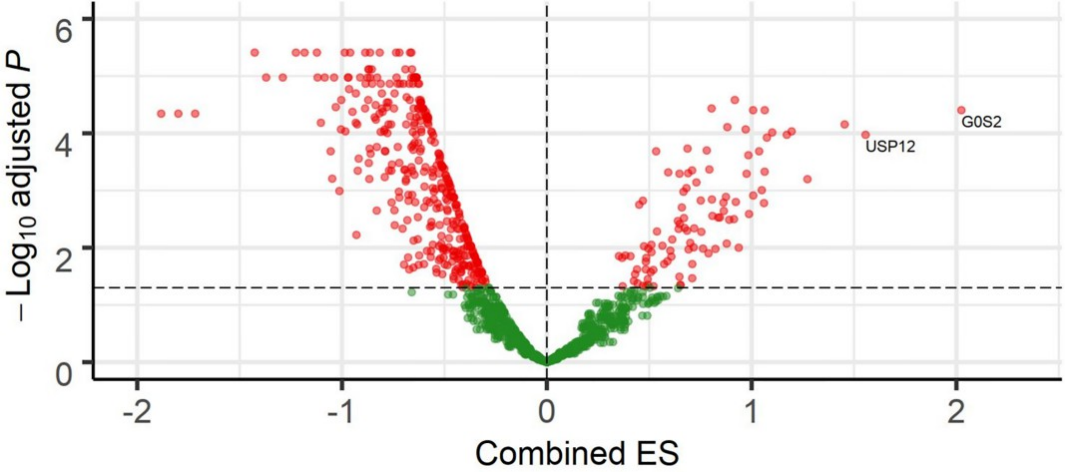
Gene expression comparison between infected and healthy controls. Volcano plot displaying combined effect size (x-axis) and negative log10 of the false discovery rate value (y-axis). The significant up and down-regulated genes are plotted as red dots.

Meta-analysis was performed on eight studies comprising 89 infected and 52 healthy human samples (Table A in S1 Data) based on the selected inclusion criteria. Before the meta-analysis, considering the platform type, the raw expression data of each study were pre-processed using a normalization method. The batch effect was also corrected using the Bayesian ComBat procedure. The processed data of each dataset were grouped into the infected and healthy conditions, and differentially expressed genes (DEGs) were identified based on Random Effect Modelling and Effect Size statistical analysis. The results of the meta-analysis showed a list of 703 DEGs (FDR <0.05), including 99 up-regulated and 604 down-regulated DEGs (Fig 2 and Table B in S1 Data). Among the DEGs, *G0S2* (ENSG00000123689), *USP12* (ENSG00000152484), and *ADM* (ENSG00000148926) were the most strongly up-regulated, while *KLHDC3* (ENSG00000124702), *FLI1* (ENSG00000151702), and *FIG 4* (ENSG00000112367) were the most strongly down-regulated in infected condition (Table B in S1 Data).

### Functional annotation

**Fig 3:**
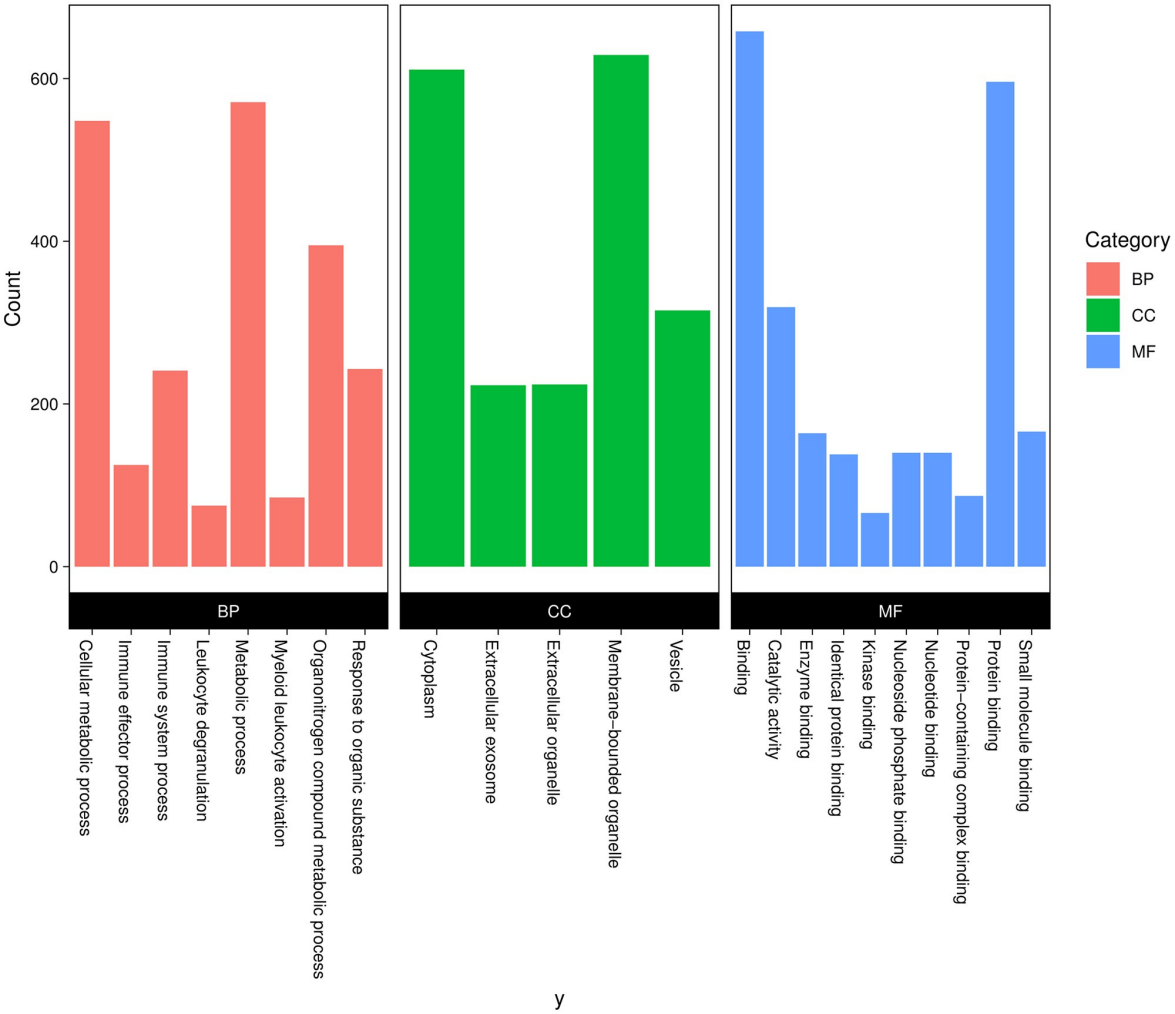
The top significant Gene Ontology (GO) categories of the differentially expressed genes (DEGs) in three ontologies: BP, biological process; MF, molecular function; and CC, cellular component.

Gene Ontology (GO) analysis was performed using the g:Profiler tool to better interpret the functional roles of the identified DEGs. In addition, the list of GO terms was reduced from 608 to 300 by REVIGO analysis. Results for enriched biological processes showed that the terms associated with the immune system, cellular metabolic process, and response to the organic substance were significantly over-represented (Fig 3 and Table C in S1 Data). Interestingly, 125 and 77 DEGs were involved in the immune effector process and cytokine production, respectively. Additionally, more than 20% of the DEGs participated in cell death and apoptotic process. In the category of molecular function, genes involved in protein binding and enzyme binding were the most enriched categories. In the category of cellular component, the DEGs were significantly enriched in the cytoplasmand extracellular exosome terms (Fig 3). The investigation conducted by GO revealed that up-regulated DEGs were closely linked to important biological processes such as cellular response to chemical stimulus, cell communication, and immune system processes. Furthermore, the study highlighted that down-regulated DEGs primarily contributed to organonitrogen compound metabolic processes (Table D in S1 Data).

### Pathway enrichment analysis

**Fig 4:**
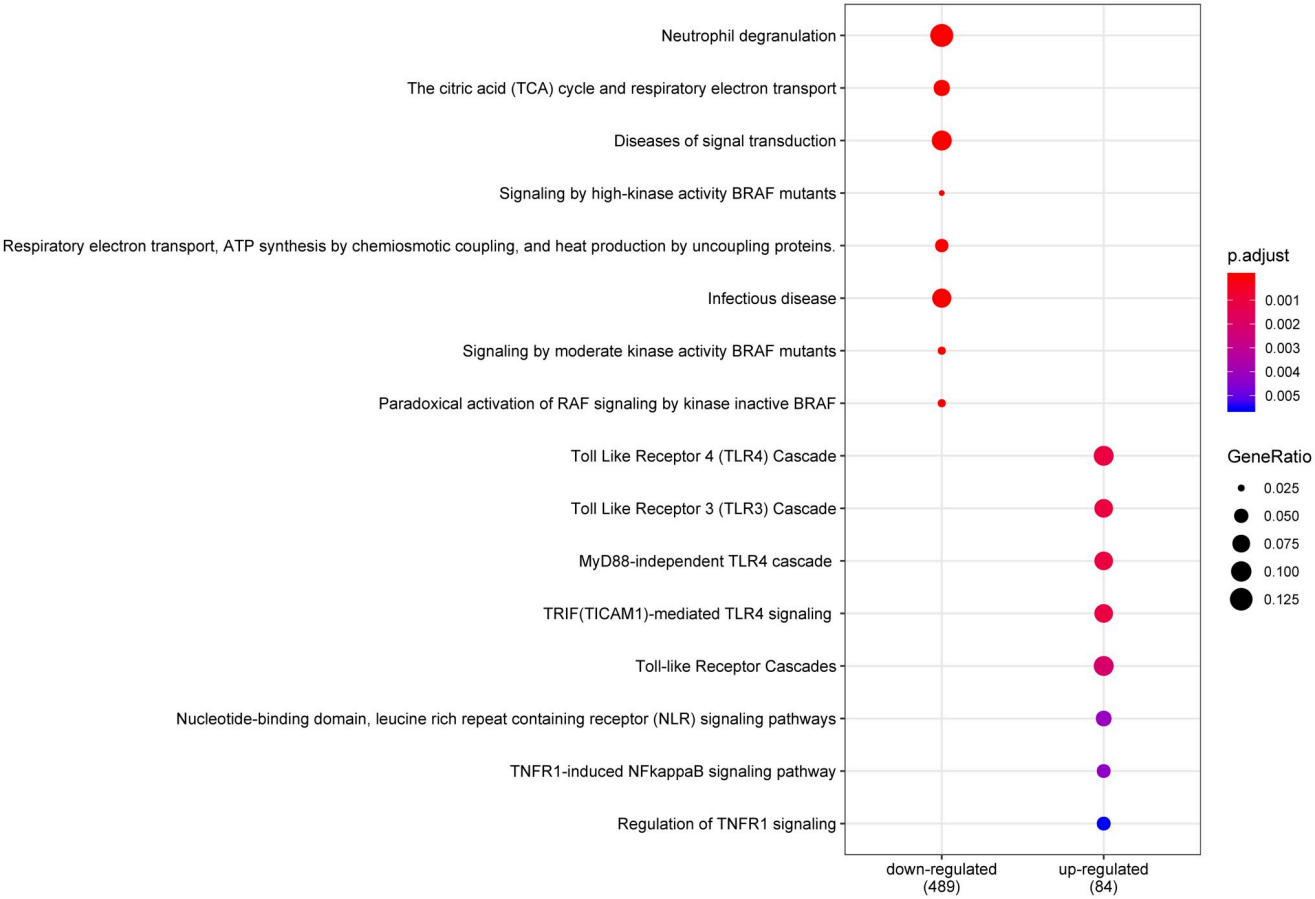
The pathway enrichment analysis of up and down-regulated genes between infected and healthy controls. The circles size and color mean the gene ratio and the adjusted p-value, respectively. The top pathways are shown.

Pathway enrichment analysis was performed based on the Reactome pathway database for the up-and down-regulated DEGs to explore biological pathways associated with the DEGs. The results showed that 489 DEGs participate in 193 pathways (Table E in S1 Data). The Toll-Like Receptor 4 (TLR4) cascade, Toll-Like Receptor 3 (TLR3) cascade, and MyD88-independent TLR4 cascade were the most significantly enriched in up-regulated genes (Fig 4 and Table E in S1 Data). Interestingly, many identified genes that regulate TNFR1 and NOD1/2 signaling were up-regulated (Table E in S1 Data). In addition, down-regulated DEGs were over-represented in neutrophil degranulation, the citric acid (TCA) cycle, and respiratory electron transport pathways (Fig 4 and Table E in S1 Data).

### Identification of transcription factors

**Fig 5:**
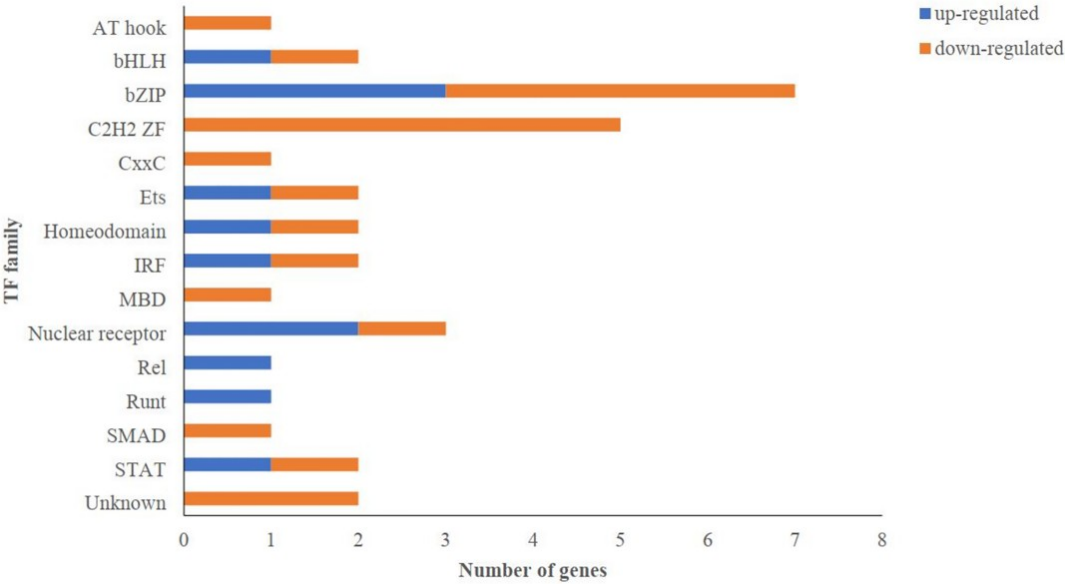
The number of differentially expressed genes (DEGs) in different transcription factor families. The number of up- or down-regulated are shown for each transcription factor family.

Transcription factors play critical roles in regulating the expression of genes related to a wide range of diseases and phenotypes [20]. Among the DEGs, 33 encoded TFs belonging to 15 families (Table F in S1 Data). Most TFs were bZIP, followed by C2H2 ZF (Fig 5). Interestingly, the expression level of most TFs in the nuclear receptor family was significantly up-regulated in infected conditions. In addition, all members of the AT-hook family were down-regulated (Fig 5).

### Weight gene co-expression network analysis

**Fig 6:**
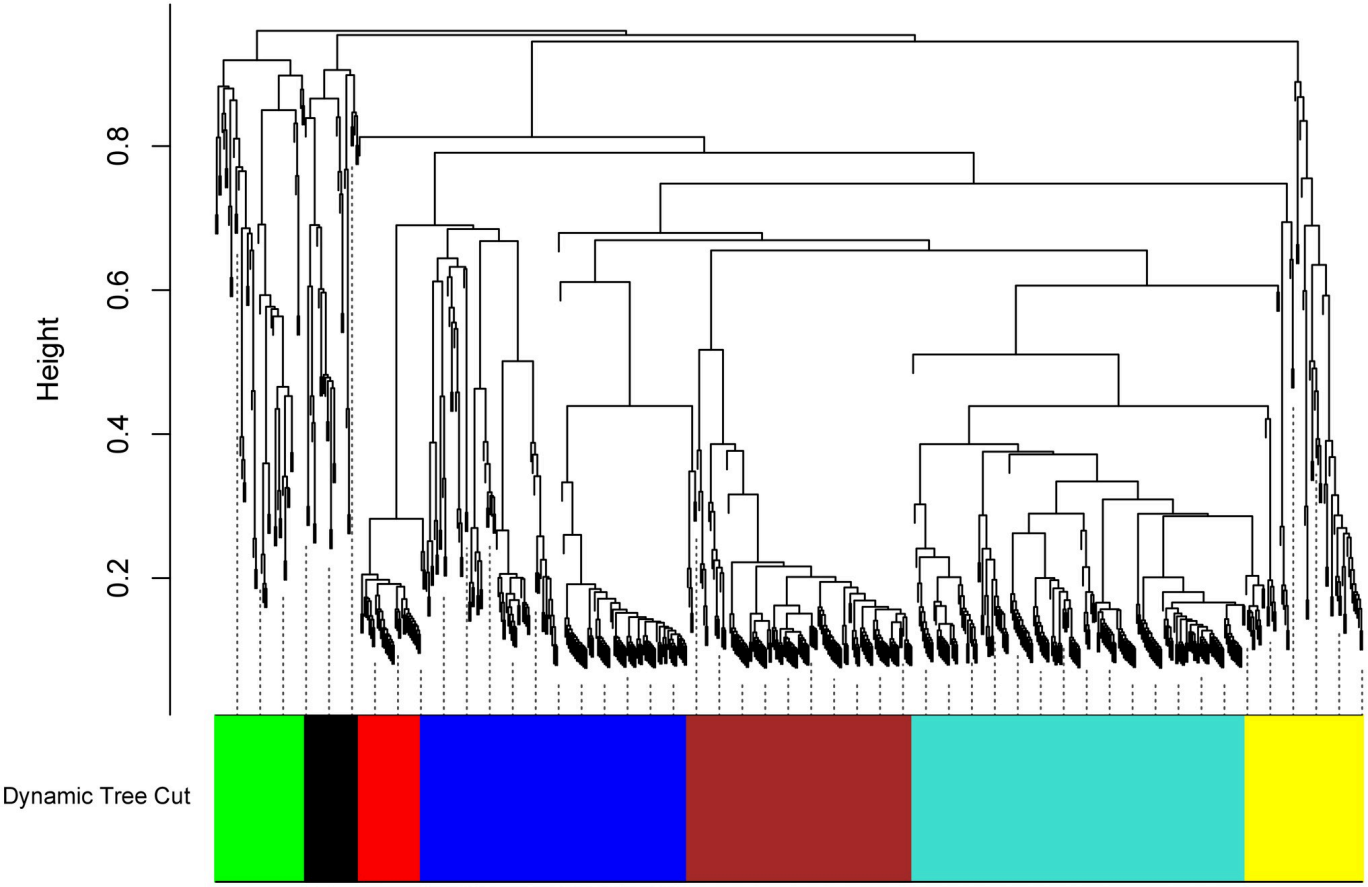
Weighted gene co-expression network analysis (WGCNA) of differentially expressed genes (DEGs). Hierarchical cluster tree representing seven modules of co-expressed genes. The gene dendrogram was constructed by clustering dissimilarity using consensus Topological Overlap. The color row indicates the corresponding module colors. Each colored row represents a module color-coded to highlight a group of genes with strong interconnections.

We performed a weighted gene co-expression network analysis (WGCNA) for the identified DEGs to obtain correlations between DEGs and construct co-expression modules. Seven distinct co-expression modules were detected, and the number of genes in these modules ranged from 33 to 204 (Fig 6). Most genes were distributed in turquoise (204) and brown (138) modules. The GO enrichment analysis was performed for modules originating from WGCNA. The GO term neutrophil degranulation and activation involved in immune response were significantly enriched for the turquoise module. Translational initiation and cellular amino acid metabolic process were significantly enriched for the brown module. In the green module, the genes were enriched in leukocyte aggregation and innate immune response. Protein deubiquitination and response to interleukin-1 were highly represented in the yellow module. The black module is highly enriched for genes involved in neutrophil degranulation and aerobic respiration. The blue module was enriched in oxidative phosphorylation and purine nucleoside triphosphate metabolic process. The blue module also included genes that are related to the regulation of autophagy (Table G in S1 Data). The red module was not enriched for any biological process terms.

### Hub gene identification in modules

Intramodular connectivity (kME) was calculated for each gene in modules to define hub genes as crucial components of the biological processes. *EIF3H* and *TMEM59* in the turquoise module, *BSG* and *SDHB* in the brown module, *DIAPH1* and *CFL1* in the blue module, *PFKFB3* and *OPTN* in the black module, *ATF3* and *TM9SF4* in the yellow module, *GOT2* and *PPP1R7* in the red module, *BIRC3* and *TRB1* in the green module exhibited a higher degree of connectivity and were identified as hub genes (Table H in S1 Data).

### Feature gene selection

As part of machine learning approaches, feature selection techniques have been widely used for high-dimensional expression data, such as select marker genes of cancer [28]. To screen the feature genes and transcriptomic signature of *Leishmania* infected cells, nine feature selection models were implemented on the gene expression dataset in infected and healthy groups. Furthermore, the accuracy evaluation of the feature-selected datasets indicated that the models generally exhibited high levels of accuracy across all algorithms (Table I in S1 Data). The results revealed that 72 genes were identified as feature genes by more than three weighting algorithms that could discriminate between infected and healthy samples (Table J in S1 Data). *G0S2* (ENSG00000123689) and *CXCL8* (ENSG00000169429) were also detected as the essential feature genes by attributed weighting algorithms. The GO analysis of feature genes revealed enrichment in biological processes such as response to stress and defense response (Table K in S1 Data). In addition, a Venn diagram (S1 Fig) showed that 54 genes selected with the feature selection models were the same as those identified with the meta-analysis approach (Table L in S1 Data), which enriched with response to organic substance and innate immune response (Table M in S1 Data).

## Discussion

The immune system’s response to *Leishmania* infection is a fascinating model of the complex interplay between pathogens and host cells. The response of immune system cells to *Leishmania* infection is crucial in determining the outcome of infection. Proper activation and regulation of these cells can lead to effective control of the parasite and prevention of disease progression. Therefore, investigating the response of immune system cells to *Leishmania* is crucial for understanding the pathogenesis of leishmaniasis. This study utilized different approaches to identify important aspects of the immune response to *Leishmania* infection. Accordingly, we identified 703 DEGs by a meta-analysis that were consistently differentially expressed in infected compared to healthy conditions. Among the top-up-regulated DEGs, *G0S2*, *USP12*, and *ADM* had the highest combined ES value (Fig 2 and Table B in S1 Data). *G0S2* is a crucial gene that encodes a small basic protein of 103 amino acids in size, regulating fat metabolism [29, 30]. It inhibits the initial step of triacylglycerol hydrolysis in adipocytes catalyzed by the adipose triglyceride lipase (ATGL) [29]. Moreover, this protein has potential roles in proliferation, apoptosis, inflammation, metabolism, and carcinogenesis, according to the previous investigation [31]. Mutations in ATGL or its inhibition result in lipolysis restriction, ultimately culminating in triglyceride accumulation [31]. Previous findings suggested measuring triglyceride as a prediction marker for VL severity since hypertriglyceridemia was a factor connected to the progression and severity of VL [32, 33]. The ubiquitin-specific protease 12 (*USP12*) as one type of deubiquitinase. Ubiquitination, which plays a role in maintaining protein balance in eukaryotic cells, is a posttranslational protein regulation mechanism and is highly conserved during evolution [34]. The prior study revealed that *Leishmania braziliensis* infection up-regulated the expression of the *USP12* in macrophage cells [35]. Adrenomedullin (ADM) is a 52-amino acid peptide that was initially isolated from human pheochromocytomas with structural similarities to calcitonin gene-related peptide (CGRP) [36]. The calcitonin-receptor-like receptor (CRLR), which has seven transmembrane domains, can operate as a receptor for CGRP and ADM depending on which members of a new family of single-transmembrane-domain proteins called receptor-activity-modifying proteins, or RAMPs, are produced [37]. ADM is also a potent vasodilator that enhances blood flow by relaxing resistance vessels [37]. Previous studies raise the possibility that RAMP2 and 3 homologs may function as regulators of CGRP and ADM activity in *Leishmania* infection [38, 39]. In addition, our results demonstrated that the top down-regulated gene was *KLHDC3* (Table B in S1 Data), one of the most significant evolutionary conserved families and associated with various proteins containing multiple kelch motifs. This gene involves many cellular functions, particularly in actin-based cytoskeleton formation, protein degradation, cell migration and transcriptional regulation [40, 41].

Furthermore, it has been discovered that kelch-repeat proteins influence the cAMP signaling system by controlling Ras and Protein Kinase [42]. In addition, within the set of DEGs, two members belonging to the STAT (signal transducer and activator of transcription) family were also detected (Table B in S1 Data). The role and function of STAT genes in patients with leishmaniasis can be situated within the host’s immunological response framework and the reciprocal interaction between the parasite and the host’s immune system. The STAT genes are involved in regulating the immune response. STATs are transcription factors that play a crucial role in mediating signaling downstream of cytokine receptors and are essential for influencing the host immune response during *Leishmania* infection [43]. Also, IFN-γ activates the JAK-STAT signaling pathway, leading to the phosphorylation of STAT proteins. These activated STAT proteins then translocate to the nucleus and bind to specific DNA sequences, leading to gene expression promoting an anti-parasitic immune response. Leishmaniasis can manifest in various forms, from localized cutaneous lesions to severe visceral disease. The type of immune response mounted against the parasite influences the disease outcome. STAT proteins can affect the severity and course of the disease by modulating the balance of pro-inflammatory and anti-inflammatory responses [43]. Due to their significant role in immunological responses, STAT proteins have been identified as potential targets for therapeutic interventions against infections such as leishmaniasis. Modulating STAT signaling could be a strategy to enhance the host’s ability to control the infection [44].

According to feature selection models, *G0S2* and *CXCL8* were detected as the most important feature genes (Table I in S1 Data). The immune response and treatment for human leishmaniasis are significantly mediated by chemokines and their receptors [45]. *CXCL8* is a chemokine secreted by tissue-resident macrophages. It acts as a chemotactic factor and induces neutrophil migration to the site of inflammation [45–47]. The results also demonstrated that 54 genes (S1 Fig) were found as significant genes by both methods, that many are associated with the immune system and TNF signaling pathway. Studies have shown that the TNF signaling pathways trigger an inflammatory response and mediate resistance to infection by *L*. *major* [48, 49]. Moreover, various genes associated with defense response, including *IRF1*, have been pinpointed (Table M in S1 Data). *IRF1* significantly influences IFN-ɣ, a vital cytokine for managing *Leishmania* infection. IFN-ɣ is produced by activated T cells and natural killer (NK) cells and plays a crucial function in activating macrophages, the primary host cells for *Leishmania* parasites. IFN-ɣ increases macrophages’ ability to eliminate intracellular parasites, known as "macrophage activation." *IRF1* contributes to the polarization of the immune response toward a Th1 profile, which is essential for efficient infection control. It affects the expression of chemokines, molecules that attract immune cells to the site of the infection. Additionally, it has been observed that *IRF1* plays a significant role in the modulation of Major Histocompatibility Complex (MHC) class I molecules. The presence of these molecules is crucial for facilitating the presentation of parasite-derived antigens to cytotoxic T cells. *IRF1* can regulate the balance between pro-inflammatory and anti-inflammatory cytokines. This regulation is essential for preventing excessive inflammation and sustaining an effective immune response against the parasite [50, 51]. Notably, we found *NCKAP1L* (ENSG00000123338), *P2RX7* (ENSG00000089041), *P2RX4* (ENSG00000135124), *JUN* (ENSG00000177606), *ADCY7* (ENSG00000121281) and *ADM* (ENSG00000148926) genes that related to *Leishmania* infection pathway among the shared genes. The Nck-associated protein 1-like (*NCKAP1L*) gene encodes a hematopoietic lineage-specific regulator of the actin cytoskeleton and is required for effective T- lymphocyte, phagocytosis, and neutrophil migration. A previous study has shown that mutations in *NCKAP1L* that abolished protein expression caused immunodeficiency, lymphoproliferation, and hyperinflammation in two human cases [52]. *P2RX4* and *P2RX7* receptors (P2X Purinoceptor4 and 7) are ligand-gated ion channels with multiple functions in immunological and central nervous system physiology. These are members of the family of ATP-binding purinoceptors [53]. These receptors function as a ligand-gated ion channel and are responsible for the ATP-dependent lysis of macrophages by developing large-molecule-permeable membrane pores. P2X4 and 7 receptors induce chemotaxis, leukocyte migration, and cytokine secretion in several models of inflammatory response. The P2X4 and 7 receptors have been implicated in controlling infection by intracellular microorganisms via a Th1/Th17 immune response. Thorstenberg et al. suggest that the IL-1β and IL-17 production were observed in UTP-treated mice by P2X4 and 7 activations during the *Leishmania amazonensis* infection [54]. At the site of infection, IL-1β and IL-17 trigger the recruitment of immune cells and microbial responses (such as ROS and NO production) that are detrimental to the *Leishmania* parasite [55]. JUN gene family (C-Jun and C-Fos) are components of the early response transcription factor AP-1, activated by various extracellular stimuli, including peptide growth factors, pro-inflammatory cytokines, and oxidative and other cellular stress [56, 57]. One of the target genes for amyloid precursor protein (APP) as an upstream regulator includes the JUN gene. A feature of pathogenesis in leishmaniasis has long been believed to involve systemic disruption of the amyloid pathway [58]. According to the pathway analysis, DEGs were targeted to TLR signaling cascades (Table E in S1 Data). TLRs are cellular receptors that participate in the innate immune system by recognizing pathogen-associated molecules [59]. It has been shown that expression of *TLR2* and *TLR4* increases during early *Leishmania* infection [60]. A large number of DEGs were also assigned to the MAPK family signaling cascades, suggesting that MAPKs may play a significant function in *Leishmania* infections. The meta-analysis data also revealed that the expression of 33 transcription factors from various families was significantly altered in infected conditions (Fig 5). The bZIP and C2H2 ZF were top TF families with greater than four genes. These TFs are mainly involved in tumorigenesis and development. The CEBPB (ENSG00000172216) TF, a member of the bZIP family, is related to the main immune response processes. The expression of *CEBPB* gene up-regulated in response to *Leishmania* infection, as previously reported [61]. The CTCF (ENSG00000102974) (a C2H2 ZF TF) mediates the macrophage function of mice infected with *Leishmania major* [62].

To explore the association between DEGs and identify hub genes, a co-expression network was constructed using the WGCNA method. The seven gene modules were obtained using WGCNA (Fig 6). Overall, modules were associated with neutrophil activation and immune response. Gene ontology analysis showed that genes in the turquoise mainly participated in neutrophil-mediated immunity (Table G in S1 Data). Neutrophils have essential roles in protection against microbial infections such as *Leishmania* infection. Several studies have reported that anti-neutrophil treatment increases the severity of leishmaniasis caused by *L*. *infantum* and *L*. *donovani* [63–65]. In addition, blue module genes are involved in oxidative phosphorylation, and yellow module genes are related to interleukin and protein deubiquitination. Studies have shown that the metabolism of M2 macrophages is based on glucose oxidative phosphorylation, fatty acid oxidation, and enhanced glutamine utilization to support their long-term wound healing and parasite defense activities. *Leishmania*-infected macrophages selectively upregulate oxidative phosphorylation, and host cells and the parasite may be involved [66]. Oxidative phosphorylation was activated after *Leishmania* infection of macrophages, which is also consistent with these macrophages’ lack of inflammatory cytokine release [67]. Numerous physiological functions, such as DNA repair, endocytic trafficking, and proteasomal degradation, are regulated by ubiquitination [68]. Several studies have investigated the importance of deubiquitinating enzymes (DUBs) and the parasite proteasome at different stages of the *Leishmania* life cycle [69–71]. The promastigote to amastigote transition has been revealed to require both ubiquitination and deubiquitination enzymes. *Leishmania major* has two ubiquitin-activating enzymes [72], and *Leishmania mexicana* has at least 20 cysteine peptidase DUBs, several of which are necessary for the promastigote to amastigote transition. The *L*. *major* Atg8 and Atg12 Ubiquitin-like modifiers have been shown to play a role in parasite autophagy [72]. Subsequently, we identified hub genes in the co-expression modules to prioritize the most critical genes by evaluating intramodular connectivity.

The enrichment analysis indicated that hub genes were mainly enriched in the C-type lectin receptor signaling pathway, HIF-1 signaling pathway, and vesicle-mediated transport. The C-type lectins (CTLs), a superfamily of glycan-binding receptors, are essential for higher animals and humans to maintain immunological homeostasis and host defense against infections [73]. The initial contact and stable interaction between the promastigote forms of the parasite and host cells is the first significant event for *Leishmania* infection. Several molecules, such as CR1, CR3, fibronectin receptors, and C-type lectin receptors expressed on the surface of macrophages, can be involved in the recognition and phagocytosis of promastigotes [74]. In macrophages and dendritic cells, the C-type lectin receptor plays a significant role in *Leishmania* sp. promastigote attachment and entrance [75]. The hypoxia-inducible factors (HIFs) are transcription factors that become activated in response to a reduction in oxygen availability. Low oxygen levels within inflammatory tissue are a common feature of *Leishmania* infection, which can increase the severity of the disease [76]. Decreased oxygen availability in hypoxic environments induces many transcription factors, including hypoxia-inducible factors (HIF-1α and HIF-21α) [77]. Leishmanicidal macrophage activity was stimulated by HIF-1 activation in response to inflammatory signals, whereas HIF-1 stabilization in the absence of inflammatory signals did not affect parasite killing [78]. During cutaneous leishmaniasis, lesions from human patients contain elevated levels of HIF-1α and the HIF target Vegfa [79].

Moreover, the *PFKFB3*, *DIAPH1*, *BSG*, *BIRC3*, *GOT2*, *EIF3H*, and *ATF3* genes were identified as module top hub genes (Table H in S1 Data). These genes are involved in glucose metabolism, signaling pathways, and apoptosis which are crucial for the immune response against parasites. In particular, The *PFKFB3* encodes the proteins involved in the synthesis and degradation of fructose-2,6-bisphosphate as a regulator molecule of glycolysis. This protein is essential for cell cycle progression and apoptosis inhibition. It has been shown that the transcription of this gene and, subsequently, glycolysis is significantly increased in *Leishmania* infection [80]. *DIAPH1* has roles in cell adhesion, motility, vesicular trafficking, and cytokinesis [81]. Meanwhile, *BIRC3* and *ATF3* are involved in apoptosis and immunity, respectively. The change of ATF3 expression is associated with generating the anti-inflammatory milieu required for *Leishmania* [82].

In conclusion, we generated a large-scale transcriptomic dataset to understand the response of immune system cells to *Leishmania* infection. We employed meta-analysis and feature selection algorithms combined with co-expression analysis that could lead to the identification of the genes, processes, and pathways that can play essential roles in leishmaniasis. We found hub genes such as *PFKFB3*, *DIAPH1*, *BSG*, *BIRC3*, *GOT2*, *EIF3H*, and *ATF3* which might serve a vital function in the response of infected cells to *Leishmania*. These hub genes are associated with crucial pathways, including the C-type lectin receptor signaling pathway and the HIF-1 signaling pathway, which are significant in maintaining immunological homeostasis and host defense against infections. In particular, the results propose that G0S2 and CXCL8 can be potential biomarkers and therapeutic targets for leishmaniasis. The findings of this study can provide valuable insights into the mechanisms of the immune system’s response to *Leishmania* infection. These insights may be utilized to identify critical targets for developing effective treatments for this disease.

## Supporting information

S1 Fig Venn diagram for overlap visualization between meta-analysis results and feature genes identified from the feature selection models. (TIF)

S1 Data The xlsx file, including the A to M Tables. (XLSX)
