# Supplementary figures and images for: Using meta-analysis and machine learning to investigate the transcriptional response of immune cells to Leishmania infection
Source: PLoS Negl Trop Dis. 2024 Jan 8;18(1):e0011892. doi: 10.1371/journal.pntd.0011892 (PMC10798641; doi:10.1371/journal.pntd.0011892)

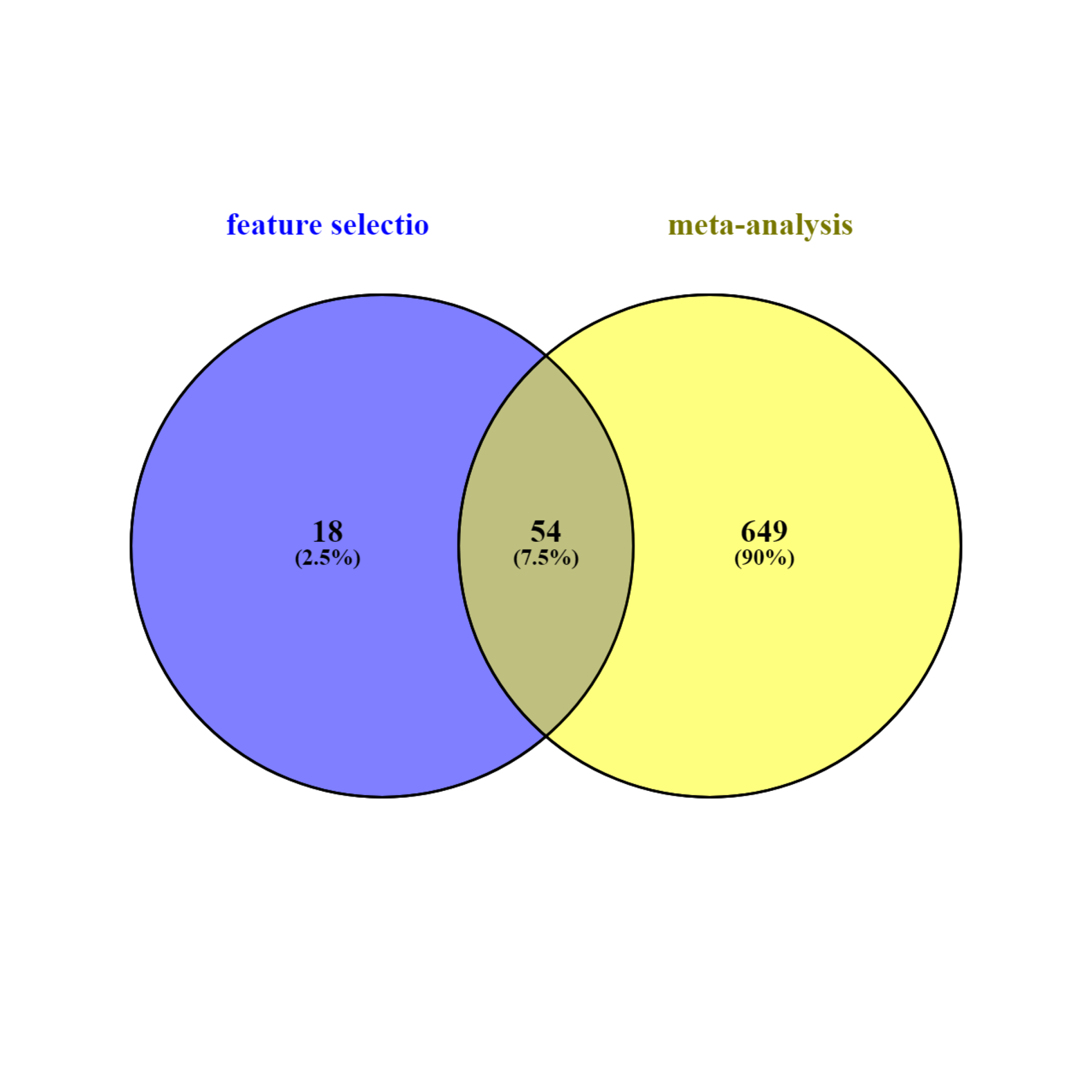

Supplement: S1 Fig — (TIF) [file pntd.0011892.s001.tif]
